# Securing Heterogeneous Wireless Sensor Networks: Breaking and Fixing a Three-Factor Authentication Protocol

**DOI:** 10.3390/s18113663

**Published:** 2018-10-29

**Authors:** Seyed Farhad Aghili, Hamid Mala, Pedro Peris-Lopez

**Affiliations:** 1 Department of Information Technology Engineering, Faculty of Computer Engineering, University of Isfahan, Hezar Jerib St., Isfahan 81746-73441, Iran; sf.aghili@eng.ui.ac.ir; 2 Department of Computer Science, University Carlos III of Madrid, Avda. de la Universidad 30, 28911 Leganés, Spain; pperis@inf.uc3m.es

**Keywords:** heterogeneous wireless sensor networks, authentication, traceability attack, de-synchronization attack

## Abstract

Heterogeneous wireless sensor networks (HWSNs) are employed in many real-time applications, such as Internet of sensors (IoS), Internet of vehicles (IoV), healthcare monitoring, and so on. As wireless sensor nodes have constrained computing, storage and communication capabilities, designing energy-efficient authentication protocols is a very important issue in wireless sensor network security. Recently, Amin et al. presented an untraceable and anonymous three-factor authentication (3FA) scheme for HWSNs and argued that their protocol is efficient and can withstand the common security threats in this sort of networks. In this article, we show how their protocol is not immune to user impersonation, de-synchronization and traceability attacks. In addition, an adversary can disclose session key under the typical assumption that sensors are not tamper-resistant. To overcome these drawbacks, we improve the Amin et al.’s protocol. First, we informally show that our improved scheme is secure against the most common attacks in HWSNs in which the attacks against Amin et al.’s protocol are part of them. Moreover, we verify formally our proposed protocol using the BAN logic. Compared with the Amin et al.’s scheme, the proposed protocol is both more efficient and more secure to be employed which renders the proposal suitable for HWSN networks.

## 1. Introduction

**Figure 1:**
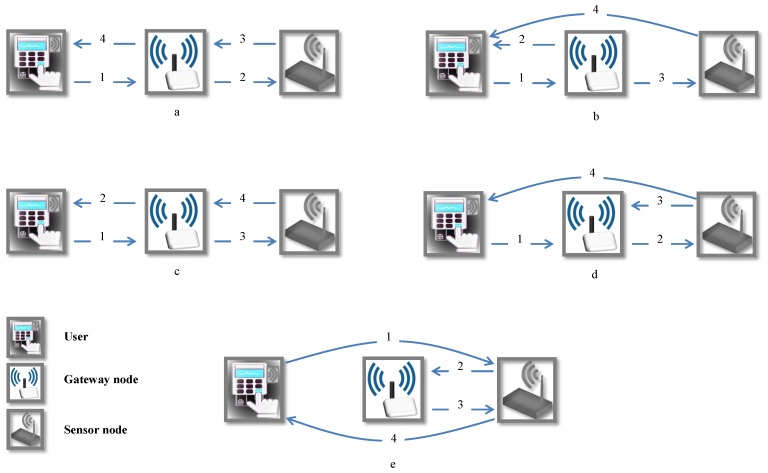
Five user authentication models in HWSN [7].

In wireless sensor networks (WSNs) there are many sensor nodes scattered in a defined area [1]. These networks can be categorized into two important classes: homogeneous and heterogeneous sensor networks. On the one hand, in homogeneous sensor networks, all the sensor nodes are equal in terms of energy and hardware complexity. On the other hand, heterogeneous sensor networks (HWSNs) include various types of wireless sensor nodes with different capabilities and functions. In HWSNs, the sensors share their functions and increase the reliability of the network without increasing the cost of implementation [2,3,4,5]. Some of these sensors are low-cost, low-power and consequently have constrained computational power, transmission range, storage capacity and battery life [6]. It is clear that there are great needs to design energy-efficient protocols for such networks. In HWSN, users communicate to the sensor nodes to acquire data of their own interest. Therefore, the user and sensor node authentication is an important line of research in HWSN security which has recently awakened interest from the network security research community. In HWSN, the gateway node (GWN) plays an essential part in the authorization procedure since this element is the connection (input/output) with the all the elements outside the network. As shown in Figure 1, there are five models in authenticating users and sensor nodes in HWSN [7]. In these five schemes, a user, a gateway node and a sensor node implement the authentication protocol by exchanging four messages (e.g., Figure 1(a.1–a.4)). In each scheme, there are four steps: (1) the gateway node authenticates the user (e.g., Figure 1(a.1)); (2) the sensor node authenticates the legitimate user and the gateway node (e.g., Figure 1(a.2)); (3) the sensor node verifies the legitimacy of the gateway node (e.g., Figure 1(a.3)); and finally, (4) in the last step, the user authenticates the legitimate sensor node (e.g., Figure 1(a.4)). Since HWSN nodes face with to many limitations in power consumption and communication range, models, in which a user and a sensor are a long way apart, are not practical, Figure 1e,b,d [8,9].

To tackle with security challenges of HWSN networks, we need lightweight enough and secure schemes. In the literature, authentication protocols are the most common adopted solution [7,10,11,12,13,14]. Unfortunately, most of them do not provide the required security and present important security pitfalls or are not energy-efficient. In this vein, recently, Amin et al. presented an untraceable and anonymous 3FA scheme for HWSNs. They used the model depicted in Figure 1a to design their protocol and asserted that their protocol can resist all common attacks known in the context of HWSN [15]. Nevertheless, in this article, we cryptanalyzed this protocol to show that this scheme is vulnerable against user impersonation, de-synchronization and session key disclosure attacks and also the adversary can trace the user. In order to hinder these attacks, we improve the Amin et al.’s protocol.

### 1.1. Our Contribution

The contributions of this article are summarized as below:

- At first, we present several serious security attacks against the Amin et al.’s scheme [15]. Our proposed attacks include de-synchronization, user impersonation, user traceability and session disclosure attacks.
- In order to increase the security level offered by Amin et al.’s protocol, we remedy the security faults found in their scheme.
- The security of the proposed scheme has be scrutinized from a formal and informal point of view. The attacks mentioned in Amin et al.’s protocol and other common security attacks have been considered in the design of the new protocol.
- The efficiency of our proposal is higher than the offered by Amin et al.’s scheme. Therefore, our scheme can be used for resource constrained sensors as the ones employed in HWSNs.

### 1.2. Paper Organization

The organization of the article is as follows. In Section 2, some related work are presented. Section 3 introduces the required preliminaries and notations. We review Amin et al.’s protocol in Section 4. Section 5 shows the security pitfalls of this scheme. We propose the improved scheme in Section 6. Then, we discuss the security of the proposed protocol in an informally way in Section 7, while, in Section 8, a formal analysis is presented. Finally, we extract some conclusions in Section 9.

## 2. Related Work

In a wireless sensor network, to allow a legitimate user to obtain information from a target sensor, the system needs to verify the validity of user by running an authentication protocol. In this section, we briefly discuss some existing schemes that aim to increase the security level of these networks.

**Two-factor Authentication Schemes:** Several two-factor authentication (2FA) schemes have been proposed for WSN, where the login phase of these protocols is based on passwords and smartcards.

In 2006, Wong et al. [16] presented a 2FA protocol based on the use of a hash function for wireless sensor networks, but the authors in [11] found that the protocol suffers from serious security pitfalls (i.e., replay, stolen-verifier and forgery attacks). To overcome these important weaknesses, authors in [11] proposed a new 2FA protocol based on passwords and smartcards. However, this protocol also is not immune against denial of service attacks and the nodes can be compromised [17].

In 2010, to improve the [11] protocol, Chen et al. [10] presented a bilateral authentication protocol in which three entities are involved (i.e., users, sensor nodes and the gateway node). In the same year, Khan et al. [12] showed that [11] fails in the authentication and in the key updating mechanism and presented a new protocol that they claimed it hinders the mentioned attacks. Later, Vaidya et al. [18] introduced several security vulnerabilities in [10,11,12] based on the stolen smartcard assumption. Xue et al. in 2013 presented a mutual authentication protocol based on temporal credentials, which is mainly based on the use of hash functions [7]. Nevertheless, He et al. [19] showed how the above protocol [7] is not resistant against user node and sensor node impersonation attacks and proposed a new temporal-credential-based protocol to overcome these weaknesses. In addition, Mir et al. [20] compromised the security of the healthcare system designed by He et al. [21], uncovering impersonation and password disclosure attacks. In addition, Turkanovic et al. [22] presented another bilateral authentication scheme in the context of HWSNs. However, Amin and Biswas [23] examined the Turkanovic et al. scheme and identified certain security problems (e.g., offline identity and password guessing attacks) and finally claimed to remove these security pitfalls in an efficient protocol. In the same year, Farash et al. [6] showed also some security shortcomings in [22] and proposed a new lightweight protocol. In the context of lightweight cryptography, Gope et al. [24] presented a 2FA protocol with especial security features including user anonymity and forward/backward secrecy. Soon, in [25], the authors analyzed the Gope’s protocol by presenting a session key disclosure attack.

**Three-Factor Authentication Schemes:** In 2016, Amin et al. [26] pointed out how the Farash et al. protocol is susceptible to a number of attacks and proposed a new mechanism which was claimed to be resistant against these attacks. To enhance the security flaws of 2FA protocols, Amin et al. proposed a three-factor authentication (3FA) scheme based on password, smartcard and biometric trait linked to the legitimate user. However, Arasteh et al. [27] proposed replay and Denial-of-Service (DoS) attacks against Amin et al.’s scheme. In 2017, the authors in [28] presented an smartcard loss attack against Amin et al.’s 3FA protocol [26]. They also showed that the attacker can reveal the session keys in other sessions of the protocol. To overcome the security flaws of this protocol, they proposed the enhanced scheme based on the Rabin’s cryptosystem. In the same year, Jiang et al. [29] presented a solution to enhance the security of another 3FA protocol [30] that suffers from important security faults including traceability, identity guessing, offline password guessing, user impersonation and server impersonation attacks.

Chang et al. in [31] found several vulnerabilities in the Turkanovic et al. 2FA protocol [22] and presented an enhancement solution, but the scheme was shown to be vulnerable to a wide set of attacks such as traceability, information disclosure or session key attacks [15]. Eventually, Amin et al. [15] presented a new untraceable and anonymous 3FA scheme for HWSNs which was argued to be the improved version of Chang et al. scheme. Nevertheless, in this article, we scrutinize the security of this 3FA protocol and show how it is vulnerable to user impersonation, de-synchronization and session key disclosure attacks and also the adversary can trace the user. To prevent these attacks, we upgrade the Amin et al.’s protocol and analyze its security from a formal and informal perspective.

**Privacy Schemes:** In some of the protocols mentioned, the authors have stated that their schemes can preserve the user’s privacy. To do this, the user’s identifier is encoded using a dynamic identity. This anonymous identifier is used when the user communicates with the gateway node, and this information is useless for the attacker to reveal the user’s identity [24]. In detail, in schemes [7,32,33], the authors claim that their proposals preserve users’ privacy. Unfortunately, all of them fail in this purpose [24].

**Threat Model:** Our threat model mainly follows the Dolev–Yao model [34]. Therefore, the adversary can intercept, modify, delete and change any of messages transmitted over the insecure communication channel. The adversary can also execute side channel attacks and then obtain the secrets stored on the smartcard. In addition, the adversary can capture the sensors and reveal their private information stored in their memory as these devices do not have tamper protection mechanisms [24].

## 3. Preliminaries and Notations

This section first shows the notations used in this paper and then revises the proposed fuzzy extractor function for extracting the biometric parameters required for the third factor of the authentication procedure.

### 3.1. Notations

The notation used through this article is summarized in the Table 1.

### 3.2. Fuzzy Extractor

The facts that biometric tokens cannot be easily guessed, are difficult to be copied, shared and forged, and are not lost or forgotten makes biometric based authentication more preferable than traditional password based ones [35,36].

A fuzzy extractor can generate cryptography keys over noisy data. In other words, they are error tolerant. In detail, this is composed of two processes, a probabilistic algorithm GEN and a deterministic algorithm REP as described below:

1. The generation procedure (GEN): given a biometric input $B_{i}$, this probabilistic algorithm generates a secret key $\psi_{i}$ and a non-secret string $\theta_{i}$, i.e., $GEN\left(B_{i}\right)=\left(\psi_{i},\theta_{i}\right)$.
2. The reproduction procedure (REP): given the noisy input $B_{i}^{*}$ and the corresponding auxiliary string $\theta_{i}$, this algorithm is able to recover the same key $\psi_{i}$ as in the generation process, i.e., $\psi_{i}=REP\left(B_{i}^{*},\theta_{i}\right)$.

## 4. Review of Amin et al.’s Scheme

In this section, we scrutinize the security of the authentication protocol proposed by Amin et al., which is composed of nine phases: (1) pre-deployment; (2) user registration; (3) login; (4) authentication and key agreement; (5) updating; (6) post-deployment; (7) password recovery; (8) password change; and (9) smartcard revocation.

### 4.1. Pre-Deployment Phase

Firstly, the gateway node GWN chooses $X_{GWN}$ as a long-term secret key and assigns identities $SID_{j}$ to the sensor nodes $S_{j}$ (1≤j≤m for a population of *m* sensor nodes in the network). Then, the GWN calculates $f_{j}=h\left(SID_{j}∥X_{GWN}\right)$ and stores $\langle SID_{j},f_{j}\rangle$ into the memory of $S_{j}$.

### 4.2. User Registration Phase

Using a secure channel, the user $U_{i}$ executes the following steps in conjunction with the GWN.

Step 1. $U_{i}$ chooses an identity $ID_{i}$, attaches to it a personal credentials (e.g., social security number), and submits both values to the GWN.
Step 2. If the GWN does not find $ID_{i}$ in the database, it generates $r_{i}\in_{R}Z_{q}^{*}$ and calculates $MI_{i}=h\left(ID_{i}∥r_{i}\right)$ and $f_{i}=h\left(MI_{i}∥X_{GWN}\right)$. Both values ($\langle MI_{i},f_{i}\rangle$) are stored in a new smartcard $SC_{i}$ and the device is handed over to $U_{i}$.
Step 3. Once receiving the smartcard, $U_{i}$ chooses a password $PW_{i}$ and then uses a sensor device to obtain his biometric information $B_{i}$ and finally writes $\langle PW_{i},ID_{i},B_{i}\rangle$ to the $SC_{i}$.
Step 4. $SC_{i}$ uses the fuzzy extractor technique to calculate $\left(\psi_{i},\theta_{i}\right)=GEN\left(B_{i}\right)$, it then computes $A_{i}=h(ID_{i}∥PW_{i}∥\psi_{i})$, $E_{i}=\theta_{i}⊕h\left(ID_{i}∥PW_{i}\right)$, $C_{i}=f_{i}⊕h\left(PW_{i}∥\psi_{i}\right)$, $REC=PW_{i}⊕h\left(ID_{i}∥\psi_{i}\right)$, $REG_{i}=h\left(ID_{i}⊕\psi_{i}\right)$ and deletes $f_{i}$.

Finally, the smartcard contains the tuple $\langle MI_{i},C_{i},E_{i},A_{i},REC,REG_{i},GEN\left(\right),REP\left(\right),h\left(\right)\rangle$.

### 4.3. Login Phase

The user $U_{i}$ follows these steps to access the data collected by sensor $S_{j}$.

Step 1. $U_{i}$ inserts $SC_{i}$ into the terminal and then enters $ID_{i}^{\prime }$ and $PW_{i}^{\prime }$ and also uses the sensor device to imprint his biometric information $B_{i}^{\prime }$.
Step 2. $SC_{i}$ retrieves $\theta_{i}^{\prime }=E_{i}⊕h\left(ID_{i}^{\prime }∥PW_{i}^{\prime }\right)$ and computes $\psi_{i}^{\prime }=REP\left(B_{i}^{\prime },\theta_{i}^{\prime }\right)$, $f_{i}^{\prime }=C_{i}⊕h\left(PW_{i}^{\prime }∥\psi_{i}^{\prime }\right)$ and $A_{i}^{\prime }=h(ID_{i}^{\prime }∥PW_{i}^{\prime }∥\psi_{i}^{\prime })$. $SC_{i}$ verifies the correctness of $A_{i}^{\prime }$. If so, $SC_{i}$ concludes $ID_{i}^{\prime }=ID_{i}$, $PW_{i}^{\prime }=PW_{i}$ and $B_{i}^{\prime }=B_{i}$; otherwise, $SC_{i}$ denies $U_{i}$.
Step 3. $SC_{i}$ generates $K_{i}\in_{R}Z_{q}^{*}$ and computes $N_{i}=h(MI_{i}∥K_{i}∥f_{i}∥T_{1}∥SID_{j})$, $L_{i}=K_{i}⊕h(MI_{i}∥f_{i}∥T_{1})$, $P_{i}=SID_{j}⊕h\left(f_{i}∥T_{1}\right)$ and $Q_{i}=h\left(ID_{i}\right)⊕h\left(K_{i}∥T_{1}\right)$ —$T_{1}$ represents the current timestamp.

Finally, $SC_{i}$ sends the tuple $\langle MI_{i},N_{i},P_{i},Q_{i},L_{i},T_{1}\rangle$ to GWN through an insecure channel.

### 4.4. Authentication and Session Key Agreement Phase

**Figure 2:**
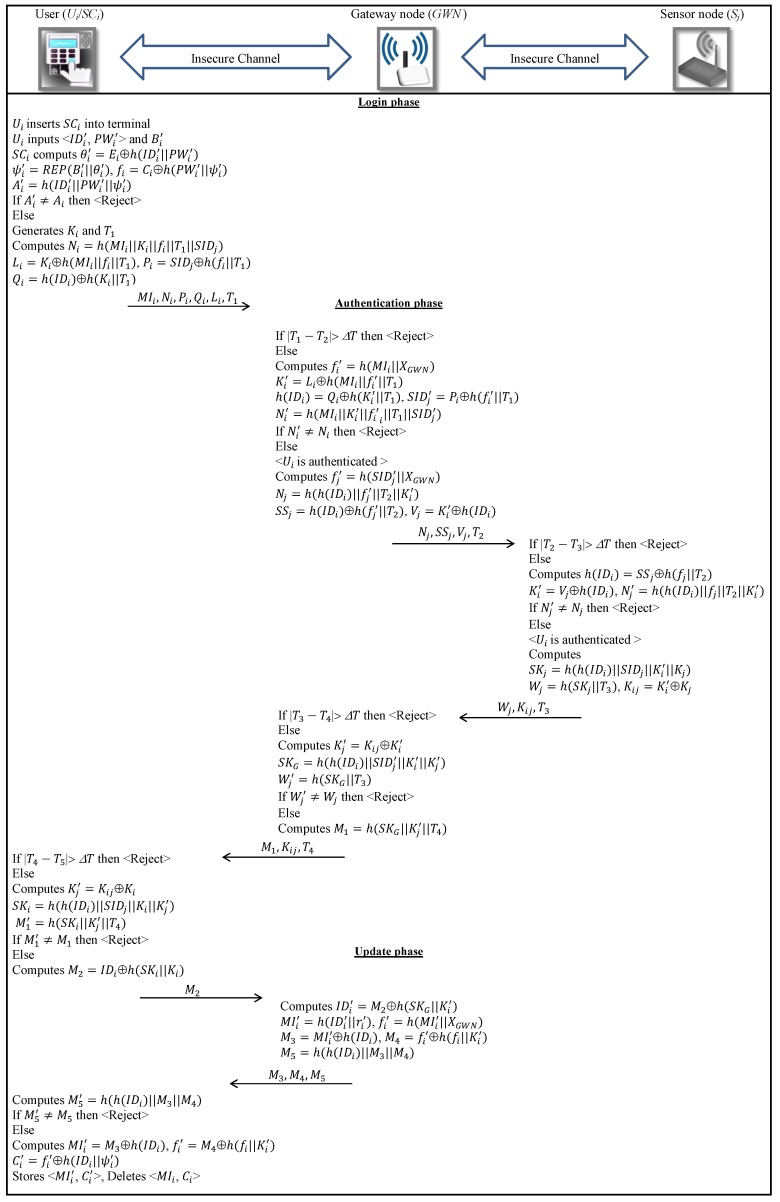
Authentication and key agreement phases in Amin et al.’s protocol [15].

Two goals are achieved in this phase (see Figure 2): (1) $U_{i}$ and $S_{j}$ are authenticated through GWN; and (2) $U_{i}$ and $S_{j}$ set a session key. In particular, the following five steps are executed.

Step 1. After receiving the message $\langle MI_{i},N_{i},P_{i},Q_{i},L_{i},T_{1}\rangle$ in login phase, the GWN checks whether the timestamp condition $|T_{1}-T_{2}|\leq \Delta T$ holds, $T_{2}$ being the current time of GWN. If the condition is fulfilled, the GWN aborts the connection. Otherwise, it calculates $f_{i}^{\prime }=h\left(MI_{i}∥X_{GWN}\right)$ and then decodes $K_{i}^{\prime }=L_{i}⊕h(MI_{i}∥f_{i}^{\prime }∥T_{1})$, $h\left(ID_{i}\right)=Q_{i}⊕h\left(K_{i}^{\prime }∥T_{1}\right)$ and $SID_{j}^{\prime }=P_{i}⊕h\left(f_{i}^{\prime }∥T_{1}\right)$. It then computes $N_{i}^{\prime }=h(MI_{i}∥K_{i}^{\prime }∥f_{i}^{\prime }∥T_{1}∥SID_{j}^{\prime })$ and checks the validity of the received $N_{i}$. If so, the GWN identifies to $U_{i}$ as an authorized user. If not, it aborts the connection.
Step 2. Then, GWN calculates $f_{j}^{\prime }=h\left(SID_{j}∥X_{GWN}\right)$, $N_{j}=h(h\left(ID_{i}\right)∥f_{j}^{\prime }∥T_{2}∥K_{i})$, $SS_{j}=h\left(ID_{i}\right)⊕h\left(f_{j}^{\prime }∥T_{2}\right)$ and $V_{j}=K_{i}⊕h\left(ID_{i}\right)$. GWN then sends the tuple $\langle N_{j},SS_{j},V_{j},T_{2}\rangle$ to $S_{j}$.
Step 3. Upon receiving the message $\langle N_{j},SS_{j},V_{j},T_{2}\rangle$, $S_{j}$ checks the validity of timestamp $T_{2}$. If $|T_{2}-T_{3}|>\Delta T$, it terminates the connection. Otherwise, $S_{j}$ computes $h\left(ID_{i}\right)=SS_{j}⊕h\left(f_{j}∥T_{2}\right)$, $K_{i}^{\prime }=V_{j}⊕h\left(ID_{i}\right)$ and the $N_{j}^{\prime }=h(h\left(ID_{i}\right)∥f_{j}∥T_{2}∥K_{i}^{\prime })$ and verifies the validity of received $N_{j}$. If it is invalid, then $S_{j}$ aborts the session. Otherwise, it generates $K_{j}\in_{R}Z_{q}^{*}$ and computes $SK_{j}=h(h\left(ID_{i}\right)∥SID_{j}∥K_{i}^{\prime }∥K_{j})$ as a session key and then computes $W_{j}=h\left(SK_{j}∥T_{3}\right)$ and $K_{ij}=K_{i}⊕K_{j}$. Then, $S_{j}$ sends the tuple $\langle W_{j},K_{ij},T_{3}\rangle$ to GWN.
Step 4. Once the message $\langle W_{j},K_{ij},T_{3}\rangle$ is received, the GWN verifies the freshness of $T_{3}$. If $|T_{3}-T_{4}|>\Delta T$, GWN aborts the connection. Otherwise, it decodes $K_{j}^{\prime }=K_{ij}⊕K_{i}$ and calculates the session key $SK_{G}=h(h\left(ID_{i}\right)∥SID_{j}^{\prime }∥K_{i}^{\prime }∥K_{j}^{\prime })$. It then computes $W_{j}^{\prime }=h\left(SK_{G}∥T_{3}\right)$ to verify the correctness of the received $W_{j}$. If the above verification fails, then GWN discontinues the session. Otherwise, it calculates $M_{1}=h(SK_{G}∥K_{j}^{\prime }∥T_{4})$ and forwards the message $\langle M_{1},K_{ij},T_{4}\rangle$ to $U_{i}$.
Step 5. Once the message $\langle M_{1},K_{ij},T_{4}\rangle$ is received, $U_{i}$ checks whether the condition $|T_{4}-T_{5}|\leq \Delta T$ is satisfied. If it is not fulfilled, $U_{i}$ aborts the session. Otherwise, it calculates $K_{j}^{\prime }=K_{ij}⊕K_{i}$, $SK_{i}=h(h\left(ID_{i}\right)∥SID_{j}∥K_{i}∥K_{j}^{\prime })$ and $M_{1}^{\prime }=h(SK_{i}∥K_{j}^{\prime }∥T_{4})$ to verify the correctness of the received $M_{1}$. Now the entities are mutually authenticated and a session key $SK_{i}=SK_{G}=SK_{j}$ has been negotiated.

### 4.5. Update Phase

In this phase, in order to achieve user untraceability, $U_{i}$ updates $\langle MI_{i},C_{i}\rangle$ as follows:

Step 1. $U_{i}$ computes $M_{2}=ID_{i}⊕h\left(SK_{i}∥K_{i}\right)$ and sends it to GWN as a confirmation message. After receiving the message, GWN decodes $ID_{i}=M_{2}⊕h\left(SK_{G}∥K_{i}^{\prime }\right)$ and updates $MI_{i}^{\prime }=h\left(ID_{i}∥r_{i}^{\prime }\right)$ and $f_{i}^{\prime }=h\left(MI_{i}^{\prime }∥X_{GWN}\right)$, where $r_{i}^{\prime }\in_{R}Z_{q}^{*}$. It then computes $M_{3}=MI_{i}^{\prime }⊕h\left(ID_{i}\right)$, $M_{4}=f_{i}^{\prime }⊕h\left(f_{i}∥K_{i}^{\prime }\right)$ and $M_{5}=h(h\left(ID_{i}\right)∥M_{3}∥M_{4})$ and sends the tuple $\langle M_{3},M_{4},M_{5}\rangle$ to $U_{i}$.
Step 2. After receiving the message $\langle M_{3},M_{4},M_{5}\rangle$, $U_{i}$ calculates $M_{5}^{\prime }=h(h\left(ID_{i}\right)∥M_{3}∥M_{4})$ to check the validity of the received $M_{5}$. If so, it extracts $MI_{i}^{\prime }=M_{3}⊕h\left(ID_{i}\right)$ and $f_{i}^{\prime }=M_{4}⊕h\left(f_{i}∥K_{i}^{\prime }\right)$ and computes $C_{i}^{\prime }=f_{i}^{\prime }⊕h\left(ID_{i}∥\psi_{i}\right)$. Then, $U_{i}$ rewrites $\langle MI_{i}^{\prime },C_{i}^{\prime }\rangle$ to $SC_{i}$ instead of previous $\langle MI_{i},C_{i}\rangle$.

### 4.6. Post-Deployment Phase

A new sensor node $S_{k}$ is used in this phase to replace a damaged sensor node $S_{j}$. The GWN generates a new identity $SID_{k}$ and then calculates $f_{k}=h\left(SID_{k}∥X_{GWN}\right)$ and stores $\langle SID_{k},f_{k}\rangle$ in $S_{k}$’s memory.

### 4.7. Password Recovery Phase

$U_{i}$ executes this phase when he forgets his password. $U_{i}$ needs to insert $SC_{i}$ in the card reader and enter his identity $ID_{i}$ along with $B_{i}$. Now, the $SC_{i}$ computes $\psi_{i}^{\prime }=REP\left(B_{i}^{\prime }∥\theta_{i}\right)$ and $REG_{i}^{\prime }=h\left(ID_{i}∥\psi_{i}^{\prime }\right)$. Then, $SC_{i}$ checks whether $REG_{i}^{\prime }=REG_{i}$. If so, then it computes $PW_{i}=REC⊕h\left(ID_{i}∥\psi_{i}\right)$ and sends the recovered password to the user.

### 4.8. Password Change Phase

The password of the user $U_{i}$ can be updated by executing the updating procedure with $SC_{i}$ and without the intervention of GWN. In detail, the following steps show how the user can update the old password $PW_{i}$ for a new one $PW_{i}^{new}$.

Step 1. $U_{i}$ inserts $SC_{i}$ in to the terminal and enters $\langle ID_{i}^{\prime },PW_{i}^{\prime }\rangle$ along with biometric information $B_{i}^{\prime }$.
Step 2. $SC_{i}$ uses the fuzzy extractor technique to calculate $\left(\psi_{i}^{\prime },\theta_{i}^{\prime }\right)=GEN\left(B_{i}^{\prime }\right)$, it then computes $A_{i}^{★}=h(ID_{i}^{\prime }∥PW_{i}^{\prime }∥\psi_{i}^{\prime })$ and $f_{i}^{\prime }=C_{i}⊕h\left(PW_{i}^{\prime }∥\psi_{i}^{\prime }\right)$. If $\left(A_{i}^{★}=A_{i}\right)$, then $SC_{i}$ requests $U_{i}$ to enter a new password $PW_{i}^{new}$ at $SC_{i}$; otherwise, $SC_{i}$ aborts this procedure.
Step 3. Now, $SC_{i}$ calculates $A_{i}^{new}=h(ID_{i}∥PW_{i}^{new}∥\psi_{i}^{\prime })$, $E_{i}^{new}=\theta_{i}^{\prime }⊕h\left(ID_{i}∥PW_{i}^{new}\right)$, $C_{i}^{new}=C_{i}⊕h\left(PW_{i}∥\psi_{i}^{\prime }⊕h\left(PW_{i}^{new}∥\psi_{i}^{\prime }\right)\right)$, $REC^{new}=PW_{i}^{new}⊕h\left(ID_{i}∥\psi_{i}^{\prime }\right)$ and replaces $\langle A_{i},E_{i},C_{i},REC\rangle$ with $\langle A_{i}^{new},E_{i}^{new},C_{i}^{new},REC^{new}\rangle$.

### 4.9. Smartcard Revocation Phase

Generally, smartcards can be lost, stolen or damaged. Thus, the smartcard revocation phase is very important. This phase is executed as described below:

Step 1. $U_{i}$ submits $ID_{i}$ and a personal credential (e.g., social security number) to the smartcard issuer.
Step 2. If the smartcard issuer can find $ID_{i}$ in the database, it generates $r_{i}\in_{R}Z_{q}^{*}$ and calculates $MI_{i}^{new}=h\left(ID_{i}∥r_{i}\right)$ and $f_{i}^{new}=h\left(MI_{i}^{new}∥X_{GWN}\right)$. It then writes $\langle MI_{i}^{new},f_{i}^{new}\rangle$ into a new smartcard $SC_{i}^{new}$ and delivers it to the user $U_{i}$.
Step 3. Once $SC_{i}^{new}$ is received, $U_{i}$ chooses a password $PW_{i}^{new}$, receives new biometric information $B_{i}^{new}$ from the sensor and writes $\langle PW_{i},ID_{i},B_{i}\rangle$ to the $SC_{i}$.
Step 4. $SC_{i}$ uses the fuzzy extractor technique to calculate $\left(\psi_{i},\theta_{i}\right)=GEN\left(B_{i}^{new}\right)$. It then computes $A_{i}^{new}=h(ID_{i}∥PW_{i}^{new}∥\psi_{i})$, $E_{i}^{new}=\theta_{i}⊕h\left(ID_{i}∥PW_{i}^{new}\right)$, $C_{i}^{new}=f_{i}^{new}⊕h\left(PW_{i}^{new}∥\psi_{i}\right)$, $REC^{new}=PW_{i}^{new}⊕h\left(ID_{i}∥\psi_{i}\right)$ and $REG_{i}^{new}=h\left(ID_{i}⊕\psi_{i}\right)$, and implants $\langle C_{i}^{new},E_{i}^{new},A_{i}^{new},REC^{new},REG_{i}^{new},GEN\left(\right),REP\left(\right),h\left(\right)\rangle$ into $SC_{i}$ and deletes $f_{i}^{new}$.

## 5. Security Analysis of Amin et al.’s Protocol

In [15], the authors claimed that the adversary/attacker *A* cannot trace or identify the user $U_{i}$ using the transmitted messages. Moreover, they claimed that the attacker cannot impersonate the user by accessing to the old login eavesdropped messages.

Unfortunately, for Amin et al.’s protocol, we show how the proposed protocol is not immune against user impersonation and de-synchronization attacks. The user can be also tracked by an attacker who eavesdrops on only one protocol session. In addition, we provide evidence of how an adversary can easily obtain the session key under the assumption that sensors are not tamper-resistant.

### 5.1. User Impersonation Attack

In this attack, we point out how an adversary *A* is authenticated by both the gateway node GWN and the sensor node $S_{j}$. The attack is described below:

- *A* eavesdrops on the message $\langle MI_{i},N_{i},P_{i},Q_{i},L_{i},T_{1}\rangle$ sent by $U_{i}$ to the GWN, then he changes the $Q_{i}$ value to $Q_{i}^{\prime }$.
- After receiving the message $\langle MI_{i},N_{i},P_{i},Q_{i}^{\prime },L_{i},T_{1}\rangle$ in the login phase, the GWN checks two issues: (1) timestamp condition $|T_{1}-T_{2}|\leq \Delta T$ and (2) validity of the received $N_{i}=h(MI_{i}∥K_{i}∥f_{i}∥T_{1}∥SID_{j})$, which does not depend on $Q_{i}$. Thus, the GWN accepts these two conditions and computes $h{\left(ID_{i}\right)}^{★}=Q_{i}^{\prime }⊕h\left(K_{i}^{\prime }∥T_{1}\right)$ and $SID_{j}^{\prime }$. It then calculates $N_{i}^{\prime }$. Now, the GWN believes that *A* is an authorized user.
- Then, GWN calculates $f_{j}^{\prime }$ and then computes $N_{j}=h(h{\left(ID_{i}\right)}^{★}∥f_{j}^{\prime }∥T_{2}∥K_{i})$, $SS_{j}=h{\left(ID_{i}\right)}^{★}⊕h\left(f_{j}^{\prime }∥T_{2}\right)$ and $V_{j}=K_{i}⊕h{\left(ID_{i}\right)}^{★}$ and sends the tuple $\langle N_{j},SS_{j},V_{j},T_{2}\rangle$ to $S_{j}$.
- $S_{j}$ check the correctness of timestamp and computes $h{\left(ID_{i}\right)}^{★}=SS_{j}⊕h\left(f_{j}∥T_{2}\right)$, $K_{i}^{\prime }=V_{j}⊕h{\left(ID_{i}\right)}^{★}$ and $N_{j}^{\prime }=h(h{\left(ID_{i}\right)}^{★}∥f_{j}∥T_{2}∥K_{i}^{\prime })$ and checks validity of the received $N_{j}$. It generates $K_{j}\in_{R}Z_{q}^{*}$ and computes $SK_{j}=h(h{\left(ID_{i}\right)}^{★}∥SID_{j}∥K_{i}^{\prime }∥K_{j})$ as a session key and then computes $W_{j}$ and $K_{ij}$. Now, the $S_{j}$ also believes that *A* is an authorized user and sends the tuple $\langle W_{j},K_{ij},T_{3}\rangle$ to GWN.
- The GWN checks the validity of $T_{3}$. It decodes $K_{j}^{\prime }$ and computes the session key $SK_{G}=h(h{\left(ID_{i}\right)}^{★}∥SID_{j}^{\prime }∥K_{i}^{\prime }∥K_{j}^{\prime })$. It then computes $W_{j}^{\prime }=h\left(SK_{G}∥T_{3}\right)$ and checks validity of the received $W_{j}$ and computes $M_{1}=h(SK_{G}∥K_{j}^{\prime }∥T_{4})$ and sends the message $\langle M_{1},K_{ij},T_{4}\rangle$ to $U_{i}$ which is the adversary. At this point, the adversary sends the random number $M_{2}$ to GWN as a confirmation message. After receiving the message, GWN uses the message to obtain $ID_{i}$ which is the random number. Due to the absence of any checking process, it employs this value to compute $M_{3}$, $M_{4}$ and $M_{5}$ and then sends the tuple $\langle M_{3},M_{4},M_{5}\rangle$ to the adversary.

Following this attack, the adversary cheats GWN and $S_{j}$ to pass the protocol with the success probability of “1”. Moreover, GWN and $S_{j}$ establish the wrong session key along with $h{\left(ID_{i}\right)}^{★}$.

### 5.2. De-Synchronization Attack

In Amin et al.’s authentication phase, an adversary *A* by eavesdropping only one session can reveal the $h(ID_{i})$ of the user $U_{i}$ and uses it to render the user to a de-synchronization state as follows. Note that, in the proposed attack, the superscript *j* indicates the parameters of the *j*-th run of protocol, j=1,2. In addition, in the Amin et al. scheme, the values of $h(ID_{i})$ of the user $U_{i}$ is a constant value. In detail, the attack can be executed following the steps described below:

- *A* eavesdrops on the message $M_{3}^{1}=MI_{i}^{2}⊕h\left(ID_{i}\right)$ from session 1;
- *A* eavesdrops on the message $MI_{i}^{2}$ from session 2;
- *A* obtains $h(ID_{i})$ from equation $h\left(ID_{i}\right)=M_{3}^{1}⊕MI_{i}^{2}$;
- In Step 6 of the authentication phase, *A* intercepts $\langle M_{3}^{2},M_{4}^{2},M_{5}^{2}\rangle$ and modifies them to $M_{3}^{★}$, $M_{4}^{★}$ and $M_{5}^{★}=h(h\left(ID_{i}\right)∥M_{3}^{★}∥M_{4}^{★})$;
- *A* sends the tuple $\langle M_{3}^{★},M_{4}^{★},M_{5}^{★}\rangle$ to $U_{i}$;
- $U_{i}$ calculates $M_{5}^{★}=h(h\left(ID_{i}\right)∥M_{3}^{★}∥M_{4}^{★})$ and then checks validity of the received $M_{5}^{★}$. Then, it extracts $MI_{i}^{\prime }=M_{3}^{★}⊕h\left(ID_{i}\right)$ and $f_{i}^{\prime }=M_{4}^{★}⊕h\left(f_{i}∥K_{i}^{\prime }\right)$ and computes $C_{i}^{\prime }=f_{i}^{\prime }⊕h\left(ID_{i}∥\psi_{i}\right)$. Then, $U_{i}$ rewrites $\langle MI_{i}^{\prime },C_{i}^{\prime }\rangle$ to $SC_{i}$ instead of previous $\langle MI_{i},C_{i}\rangle$.

Following this attack, the adversary compels the $U_{i}$ to insert the wrong $\langle MI_{i},C_{i}\rangle$ into $SC_{i}$’s memory. Now, $U_{i}$ cannot use $SC_{i}$ to do the login.

### 5.3. User Traceability Attack

Following the privacy model proposed by Ouafi and Phan [37], the attacker can perform following phases to mount a traceability attack.

Step 1. In round *n*, *A* sends an $Execute\;query(GWN,U_{0},n)$ and eavesdrops on messages $MI_{0,n}^{U_{0}}$, $Q_{0,n}^{U_{0}}=h{\left(ID_{0}\right)}_{n}^{U_{0}}⊕h\left(K_{0,n}^{U_{0}}∥T_{1,n}^{U_{0}}\right)$, $T_{1,n}^{U_{0}}$, $V_{j,n}^{S_{j}}=K_{0,n}^{U_{0}}⊕h{\left(ID_{0}\right)}_{n}^{U_{0}}$ and $M_{3,n}^{GWN}$;
Step 2. The adversary *A* selects two users $U_{0}$ and $U_{1}$ and sends a $Test\;query(U_{1},U_{0},n+1)$ and depending on the random bit b∈{0,1} the adversary *A* receives a $h{\left(ID_{b}\right)}^{U_{b}}\in \left\{h{\left(ID_{0}\right)}^{U_{0}},h{\left(ID_{1}\right)}^{U_{1}}\right\}$ corresponding to users $\left\{U_{0},U_{1}\right\}$;
Step 3. *A* sends an $Execute\;query(GWN,U_{b},n+1)$ and eavesdrops on messages $MI_{b,n+1}^{U_{b}}$, $Q_{b,n+1}^{U_{b}}=h{\left(ID_{b}\right)}_{n+1}^{U_{b}}⊕h\left(K_{b,n+1}^{U_{b}}∥T_{1,n+1}^{U_{b}}\right)$, $T_{1,n+1}^{U_{b}}$, $V_{j,n+1}^{S_{j}}=K_{b,n+1}^{U_{b}}⊕h{\left(ID_{b}\right)}_{n+1}^{U_{b}}$ and $M_{3,n+1}^{GWN}$;
Step 4. *A* guesses the random bit b=0 if $h{\left(ID_{0}\right)}_{n}^{U_{0}}=h{\left(ID_{b}\right)}_{n+1}^{U_{b}}$ with a probability higher than a random coin flip following the procedure described below.
Step 5. We have,
  $h{\left(ID_{b}\right)}_{n+1}^{U_{b}}=Q_{b,n+1}^{U_{b}}⊕h(\left(V_{j,n+1}^{S_{j}}⊕\left(MI_{b,n+1}^{U_{b}}⊕M_{3,n}^{GWN}\right)∥T_{1,n+1}^{U_{b}}\right)$,
  $h{\left(ID_{0}\right)}_{n}^{U_{0}}=Q_{0,n}^{U_{0}}⊕h(\left(V_{j,0}^{S_{j}}⊕\left(MI_{b,n+1}^{U_{b}}⊕M_{3,n}^{GWN}\right)∥T_{1,n}^{U_{0}}\right)$,
  - As $h{\left(ID_{i}\right)}^{U_{i}}$ is constant and the user does not update it,
  - If $h{\left(ID_{b}\right)}_{n+1}^{U_{b}}=h{\left(ID_{0}\right)}_{n}^{U_{0}}$, then $U_{b}=U_{0}$.
Step 6. As a result, we can express $Adv_{A}^{UNT}\left(k\right)=|Pr\left[A\;guesses\;b\;correctly\right]-\frac{1}{2}|=|1-\frac{1}{2}|=\frac{1}{2}\gg \epsilon \left(k\right)$;

Following the described attack, the attacker can trace any target user $U_{i}$. In other words, Amin et al.’s scheme is not resistant against user traceability attack.

### 5.4. Session Key Disclosure Attack

As described in Section 5.2, *A* can extract $h(ID_{i})$ belonged to $U_{i}$. Thus, if we assume that the sensor $S_{j}$ is not equipped with tamper-resistant, *A* obtains $\langle SID_{j},f_{j}\rangle$ from sensor’s memory—note that the adversary does not require $f_{j}$ to execute the proposed attack. Then, it executes the session key disclosure attack as follows:

- *A* eavesdrops on messages $T_{1}$ and $V_{j}=K_{i}^{\prime }⊕h{\left(ID_{i}\right)}^{\prime }$;
- *A* obtains $K_{i}$ from equation $K_{i}=V_{j}⊕h\left(ID_{i}\right)$;
- *A* obtains $K_{j}$ from equation $K_{j}=K_{ij}⊕K_{i}$;
- *A* computes the session key $SK_{j}$ using the $SK_{j}=h(h\left(ID_{i}\right)∥SID_{j}∥K_{i}^{\prime }∥K_{j})$.

Therefore, an adversary can disclose the session key in Amin et al.’s protocol.

Finally, we would like to highlight that all our proposed attacks exploit the fact that the bitwise XOR operation is a source of vulnerability against passive and active attacks [38,39,40].

## 6. Our Proposed Protocol

We present an enhanced version of Amin et al.’s protocol to remedy its security pitfalls. The scheme, as the original proposal, is split into night phases: (1) pre-deployment; (2) user registration; (3) login; (4) authentication and key agreement; (5) update; (6) post-deployment; (7) password recovery; (8) password change; and (9) smart revocation. As we only enhanced the (3), (4), and (5) phases, these are the ones that we describe.

**Figure 3:**
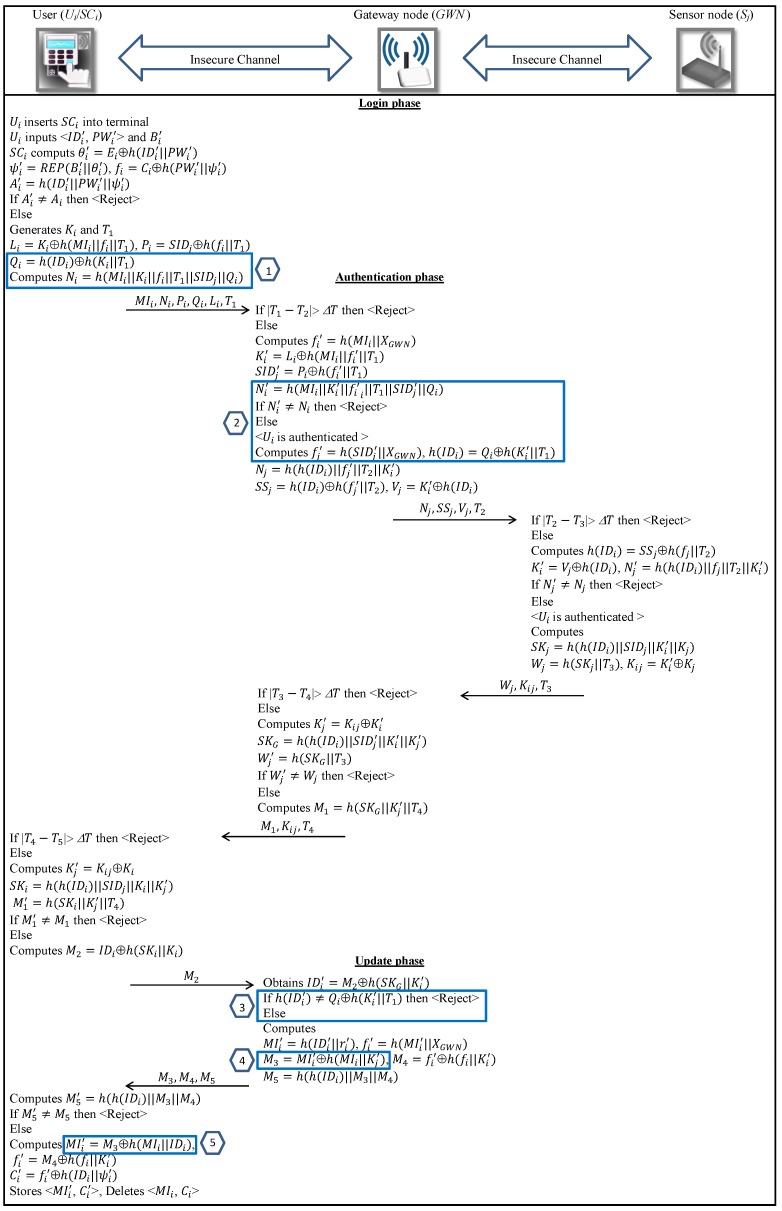
Modified Amin et al.’s authentication and key agreement phase. Changes are highlighted by boxes in the proposed scheme.

In summary, the enhanced authentication and key agreement phase, and update phase of the proposed scheme, as shown in the blue boxes in Figure 3, have five important changes. To prevent the user impersonation attack, the user makes uses of $Q_{i}$ in the message $N_{i}$. Subsequently, the gateway node GWN verifies this value to authenticate the legitimate user (boxes number 1 and 2). To overcome the de-synchronization attack, we change the format of message $M_{3}$ as well as the equation the user employs to update $MI_{i}$. Therefore, the attacker cannot obtain $h(ID_{i})$ by XORing these two values (boxes number 4 and 5). To avoid the replay attack, the gateway node GWN checks the validity of $M_{2}$ by verifying the value of $h(ID_{i})$ (box number 3).

### 6.1. Login Phase

In this phase, we employ the $Q_{i}$ in $N_{i}$ to guarantee the integrity of $Q_{i}$. $U_{i}$ performs the following steps to login when it wishes to access data collected by $S_{j}$:

Step 1. $U_{i}$ inserts $SC_{i}$ into the terminal and then enters $ID_{i}^{\prime }$ and $PW_{i}^{\prime }$ and also uses the sensor device to imprint his biometric information $B_{i}^{\prime }$.
Step 2. $SC_{i}$ retrieves $\theta_{i}^{\prime }=E_{i}⊕h\left(ID_{i}^{\prime }∥PW_{i}^{\prime }\right)$ and computes $\psi_{i}^{\prime }=REP\left(B_{i}^{\prime },\theta_{i}^{\prime }\right)$, $f_{i}^{\prime }=C_{i}⊕h\left(PW_{i}^{\prime }∥\psi_{i}^{\prime }\right)$ and $A_{i}^{\prime }=h(ID_{i}^{\prime }∥PW_{i}^{\prime }∥\psi_{i}^{\prime })$. $SC_{i}$ checks validity of $A_{i}^{\prime }$. If so, $SC_{i}$ implies $ID_{i}^{\prime }=ID_{i}$, $PW_{i}^{\prime }=PW_{i}$ and $B_{i}^{\prime }=B_{i}$; otherwise, $SC_{i}$ denies $U_{i}$.
Step 3. $SC_{i}$ generates $K_{i}\in_{R}Z_{q}^{*}$ and calculates $L_{i}=K_{i}⊕h(MI_{i}∥f_{i}∥T_{1})$, $P_{i}=SID_{j}⊕h\left(f_{i}∥T_{1}\right)$, $Q_{i}=h\left(ID_{i}\right)⊕h\left(K_{i}∥T_{1}\right)$ and $N_{i}=h(MI_{i}∥K_{i}∥f_{i}∥T_{1}∥SID_{j}∥Q_{i})$, $T_{1}$ being the current timestamp.

After this, $SC_{i}$ forwards the tuple $\langle MI_{i},N_{i},P_{i},Q_{i},L_{i},T_{1}\rangle$ to GWN using a public communication channel.

### 6.2. Authentication and Session Key Agreement Phase

At this point, $U_{i}$ and $S_{j}$ are authenticated through GWN and a session key is set between both entities. In addition, we modify the message $M_{3}$ to tackle the attacker when she tries to obtain $h(ID_{i})$ in the next session. In Figure 3, we summarize the details of this phase:

Step 1. Once the message $\langle MI_{i},N_{i},P_{i},Q_{i},L_{i},T_{1}\rangle$ is received in the Login phase, the GWN checks whether the timestamp condition $|T_{1}-T_{2}|\leq \Delta T$ holds. If the condition is fulfilled, the GWN terminates the connection. Otherwise, it calculates $f_{i}^{\prime }=h\left(MI_{i}∥X_{GWN}\right)$ and then decodes $K_{i}^{\prime }=L_{i}⊕h(MI_{i}∥f_{i}^{\prime }∥T_{1})$ and $SID_{j}^{\prime }=P_{i}⊕h\left(f_{i}^{\prime }∥T_{1}\right)$. It then calculates $N_{i}^{\prime }=h(MI_{i}∥K_{i}^{\prime }∥f_{i}^{\prime }∥T_{1}∥SID_{j}^{\prime }∥Q_{i})$ and checks validity of the received $N_{i}$. If so, the GWN identifies to $U_{i}$ as an authorized user. If not, it terminates the connection.
Step 2. Then, GWN obtains $h\left(ID_{i}\right)=Q_{i}⊕h\left(K_{i}^{\prime }∥T_{1}\right)$ and calculates $f_{j}^{\prime }=h\left(SID_{j}∥X_{GWN}\right)$ and then computes $N_{j}=h(h\left(ID_{i}\right)∥f_{j}^{\prime }∥T_{2}∥K_{i})$, $SS_{j}=h\left(ID_{i}\right)⊕h\left(f_{j}^{\prime }∥T_{2}\right)$ and $V_{j}=K_{i}⊕h\left(ID_{i}\right)$, $T_{2}$ being the current timestamp. GWN then forwards the tuple $\langle N_{j},SS_{j},V_{j},T_{2}\rangle$ to $S_{j}$.
Step 3. Once the message $\langle N_{j},SS_{j},V_{j},T_{2}\rangle$ is received, $S_{j}$ checks validity of the timestamp $T_{2}$. If $|T_{2}-T_{3}|>\Delta T$, it terminates the connection. Otherwise, $S_{j}$ calculates $h\left(ID_{i}\right)=SS_{j}⊕h\left(f_{j}∥T_{2}\right)$, $K_{i}^{\prime }=V_{j}⊕h\left(ID_{i}\right)$ and $N_{j}^{\prime }=h(h\left(ID_{i}\right)∥f_{j}∥T_{2}∥K_{i}^{\prime })$ and checks validity of the received $N_{j}$. If the verification fails, then $S_{j}$ aborts the session. Otherwise, it generates $K_{j}\in_{R}Z_{q}^{*}$ and computes $SK_{j}=h(h\left(ID_{i}\right)∥SID_{j}∥K_{i}^{\prime }∥K_{j})$ as the session key and then computes $W_{j}=h\left(SK_{j}∥T_{3}\right)$ and $K_{ij}=K_{i}⊕K_{j}$. Finally, $S_{j}$ sends the tuple $\langle W_{j},K_{ij},T_{3}\rangle$ to GWN.
Step 4. Once the message $\langle W_{j},K_{ij},T_{3}\rangle$ is received, the GWN verifies the correctness of $T_{3}$. If $|T_{3}-T_{4}|>\Delta T$, GWN aborts the connection. Otherwise, it decodes $K_{j}^{\prime }=K_{ij}⊕K_{i}$ and computes the session key $SK_{G}=h(h\left(ID_{i}\right)∥SID_{j}^{\prime }∥K_{i}^{\prime }∥K_{j}^{\prime })$. It then computes $W_{j}^{\prime }=h\left(SK_{G}∥T_{3}\right)$ and checks the validity of the received $W_{j}$. If the above verification fails, then GWN discontinues the session. Otherwise, it calculates $M_{1}=h(SK_{G}∥K_{j}^{\prime }∥T_{4})$ and forwards the message $\langle M_{1},K_{ij},T_{4}\rangle$ to $U_{i}$.
Step 5. Once the message $\langle M_{1},K_{ij},T_{4}\rangle$ is received, $U_{i}$ checks whether the condition $|T_{4}-T_{5}|\leq \Delta T$ is satisfied. If it does not fulfilled, $U_{i}$ ends the session. Otherwise, it calculates $K_{j}^{\prime }=K_{ij}⊕K_{i}$, $SK_{i}=h(h\left(ID_{i}\right)∥SID_{j}∥K_{i}∥K_{j}^{\prime })$ and $M_{1}^{\prime }=h(SK_{i}∥K_{j}^{\prime }∥T_{4})$ and checks the validity of $M_{1}$. At this point, the entities are mutually authenticated and a session key $SK_{i}=SK_{G}=SK_{j}$ has been negotiated.

### 6.3. Update Phase

In this phase, $U_{i}$ updates $\langle MI_{i},C_{i}\rangle$ in order to achieve user untraceability, as described in the next steps and depicted in Figure 3:

Step 1. $U_{i}$ computes $M_{2}=ID_{i}⊕h\left(SK_{i}∥K_{i}\right)$ and sends it to GWN as a confirmation message. After receiving the message, GWN decodes $ID_{i}=M_{2}⊕h\left(SK_{G}∥K_{i}^{\prime }\right)$ and checks if the condition $h\left(ID_{i}\right)=Q_{i}⊕h\left(K_{i}^{\prime }∥T_{1}\right)$ holds. If the verification fails, then GWN aborts the session. Otherwise, it updates $MI_{i}^{\prime }=h\left(ID_{i}∥r_{i}^{\prime }\right)$ and $f_{i}^{\prime }=h\left(MI_{i}^{\prime }∥X_{GWN}\right)$, where $r_{i}^{\prime }\in_{R}Z_{q}^{*}$. It then computes $M_{3}=MI_{i}^{\prime }⊕h\left(MI_{i}∥K_{j}^{\prime }\right)$, $M_{4}=f_{i}^{\prime }⊕h\left(f_{i}∥K_{i}^{\prime }\right)$ and $M_{5}=h(h\left(ID_{i}\right)∥M_{3}∥M_{4})$ and sends the tuple $\langle M_{3},M_{4},M_{5}\rangle$ to $U_{i}$.
Step 2. After receiving the message $\langle M_{3},M_{4},M_{5}\rangle$, $U_{i}$ calculates $M_{5}^{\prime }=h(h\left(ID_{i}\right)∥M_{3}∥M_{4})$ and then checks validity of $M_{5}$. If so, it extracts $MI_{i}^{\prime }=M_{3}⊕h\left(MI_{i}∥K_{j}^{\prime }\right)$ and $f_{i}^{\prime }=M_{4}⊕h\left(f_{i}∥K_{i}^{\prime }\right)$ and computes $C_{i}^{\prime }=f_{i}^{\prime }⊕h\left(ID_{i}∥\psi_{i}\right)$. Then, $U_{i}$ rewrites $\langle MI_{i}^{\prime },C_{i}^{\prime }\rangle$ to $SC_{i}$ instead of previous $\langle MI_{i},C_{i}\rangle$.

## 7. Security Analysis of the Proposed Protocol

The proposed protocol is analyzed from an informal and formal point of view. This analysis shows how the proposed scheme withstands relevant and common security attacks.

The informal security analysis of a security scheme discusses its robustness against the common attacks known in its context. However, the formal security analysis methods employ mathematics or logic tools such as BAN-logic [41], AVISPA [42] or Proverif [43] to formally scrutinize the security of a cryptographic protocol. In this article, we employ the BAN-logic tool to formally verify our proposed protocol.

### 7.1. Informal Security Analysis

In this section, we point out how our proposed protocol withstands against relevant and well-known attacks.

#### 7.1.1. Stolen Smartcard Attack

In our proposal, if the smartcard $SC_{i}$ is stolen or lost, the adversary can access its memory and obtain all the information $MI_{i}$, $A_{i}$, $E_{i}$, $C_{i}$, REC and $REG_{i}$ stored in the smartcard. Note that, in our protocol, the smartcard is not tamper-resistant. Since some values ($ID_{i}$, $PW_{i}$ and $B_{i}$) are unknown for the adversary, s/he cannot compute $\theta_{i}^{\prime }=E_{i}⊕h\left(ID_{i}^{\prime }∥PW_{i}^{\prime }\right)$, $\psi_{i}^{\prime }=REP\left(B_{i}^{\prime },\theta_{i}^{\prime }\right)$ and $f_{i}^{\prime }=C_{i}⊕h\left(PW_{i}^{\prime }∥\psi_{i}^{\prime }\right)$ without having any information about these parameters. Furthermore, it is also computationally unfeasible for the attacker to disclose the $ID_{i}$, $PW_{i}$ and the secret biometric information $B_{i}$ of the user $U_{i}$ thanks to the collision-resistance property of the one-way hash function. Thus, the proposed protocol is secure against the stolen smartcard attack.

#### 7.1.2. Offline Password Guessing Attack

In our scheme, the password $PW_{i}$ of the user $U_{i}$ is involved in $A_{i}$, $E_{i}$, $C_{i}$ and REC values, which are stored in the smartcard. As discussed above, the adversary *A* cannot use any of these stored items to obtain the password. In addition, using the messages transferred from the user $U_{i}$, the attacker cannot relate these messages to the items stored on the smartcard to find useful information to verify her/his guess about $PW_{i}$. Therefore, our proposed scheme is robust against offline password guessing attack.

#### 7.1.3. Privileged Insider Attack

In this kind of attack, the insider attacker tries to impersonate the legitimate user by using this user’s password. However, in the user registration phase of our scheme, $U_{i}$ only submits $ID_{i}$ as a registration request. In addition, all the messages transmitted via a public channel are independent of $ID_{i}$. Thus, by no means can the insider of GWN get $U_{i}$’s password. That is, our proposed protocol is resistant against the privileged insider attack.

#### 7.1.4. Offline Identity Guessing Attack

On this occasion, the adversary tries to obtain knowledge about the real identity $ID_{i}$ of a user $U_{i}$ —the user and GWN are the unique entities who know this information. In our proposal, the adversary cannot derive $ID_{i}$ from information obtained from the smartcard. In addition, $ID_{i}$ is never passed over the public communication channel. As a consequence of using the one-way hash function h(·), the adversary cannot find any useful information related to $ID_{i}$ to verify her/his guess. Therefore, our proposed scheme is robust against identity guessing attack.

#### 7.1.5. User Impersonation Attack

In this attack, the adversary aims to cheat GWN by attempting to take the place of a legitimate user in the logging phase. S/he may use the eavesdropped login message $\langle MI_{i},N_{i},P_{i},Q_{i},L_{i},T_{1}\rangle$ of the previous sessions to conduct her/his attack. We show how our scheme is resistant against this attack. Once the eavesdropped message is received, the GWN checks the legitimacy of the user $U_{i}$ by validating $N_{i}=h(MI_{i}∥K_{i}∥f_{i}∥T_{1}∥SID_{j}∥Q_{i})$. *A* has to possess $f_{i}$ and $h(ID_{i})$ to forge $N_{i}$. However, without having any knowledge about the password $ID_{i}$, the biometric key and the $SID_{j}$ of the smartcard, the adversary *A* cannot calculate a valid $N_{i}$. Therefore, our proposed scheme is secure against user impersonation attack.

#### 7.1.6. Gateway Node Impersonation Attack

To impersonate the gateway node, the adversary has to forge the message $\langle N_{j},SS_{j},V_{j},T_{2}\rangle$. Thus, the adversary *A* needs to know $f_{j}$, $K_{i}$ and $h(ID_{i})$ to compute $N_{j}=h(h\left(ID_{i}\right)∥f_{j}^{\prime }∥T_{2}∥K_{i}),$ which is impossible. Thus, *A* cannot forge the aforementioned message. In addition, *A* cannot compute $M_{1}=h(SK_{G}∥K_{j}^{\prime }∥T_{4})$ and $K_{ij}=K_{i}⊕K_{j}$, which are created by GWN. Therefore, our proposed scheme resists GWN impersonation attack.

#### 7.1.7. Sensor Node Impersonation Attack

In the authentication phase, the typical sensor node $S_{j}$ computes $W_{j}=h\left(SK_{j}∥T_{3}\right)$ and $K_{ij}=K_{i}⊕K_{j}$ and sends these values along with $T_{3}$ to the gateway node GWN. To forge the messages $W_{j}$ and $K_{ij}$, the adversary *A* must compute $SK_{j}=h(h\left(ID_{i}\right)∥SID_{j}∥K_{i}∥K_{j})$ and must know $K_{i}$ and $K_{j}$. Moreover, *A* cannot compute $SK_{j}$ without the knowing $h(ID_{i})$ and $SID_{j}$. Therefore, *A* cannot compute $S_{j}$’s messages to execute a sensor node impersonation attack.

#### 7.1.8. Session Key Security

In the authentication and session key agreement, the attacker can eavesdrop the messages $W_{j}=h\left(SK_{j}∥T_{3}\right)$ and $M_{1}=h(SK_{G}∥K_{j}∥T_{4})$. Nevertheless, the session key $SK_{j}=SK_{G}=h(h\left(ID_{i}\right)∥SID_{j}∥K_{i}∥K_{j})$ is protected by the usage of the one-way hash function h(·). For this, it is computationally impossible for the adversary to derive the used key. Thus, our proposed scheme provides session key security.

#### 7.1.9. User Anonymity

In our proposed protocol, the identity $ID_{i}$ of user $U_{i}$ is never passed in plain-text over an insecure communication channel. In this sense, $h(ID_{i})$ is the value transmitted in the public messages. Due to the collision-resistant property of the one-way hash function h(·), deriving $ID_{i}$ from $h(ID_{i})$ is computationally impossible for the attacker. Therefore, our proposed scheme preserves user anonymity.

#### 7.1.10. Preserving User Untraceability

In this attack, an adversary *A* aims to determine whether two messages are generated by the same (unknown) user. Luckily, in our proposal, the attacker cannot be able to find any relationship between $Q_{i}$, $M_{2}$ and user’s identity $ID_{i}$. Furthermore, it must be noted that, in our proposed protocol, all the parameters used in the messages $\langle MI_{i},N_{i},P_{i},Q_{i},L_{i},T_{1}\rangle$ are random. Moreover, when the update phase of the protocol is executed, $U_{i}$ updates $\langle MI_{i},C_{i}\rangle$ for the next session. Therefore, *A* cannot determine whether two protocol sessions are linked to the same user. Therefore, in our proposed protocol, users cannot be tracked.

#### 7.1.11. Replay Attack

In the replay attack, the adversary forwards eavesdropped messages of the protocol (previous sessions) to try to deceive legitimate entities. The timestamp values and random numbers used in all messages of the protocol prevents any replay efforts from attacker. Therefore, replay attacks can be identified by verifying the freshness of the timestamp values and random numbers. Therefore, the replay attack does not work in our scheme.

### 7.2. Formal Security Analysis

We use BAN-logic [41] to conduct the security analysis of the authentication and key agreement phase of our proposal. Table 2 summarizes the used notation. Thereupon, we introduce the two main rules used in our analysis.

**R1 (Shared key rule).**$\frac{P|\equiv P\overset{k}{⟷}Q,P◃{\left[X\right]}_{k}}{P|\equiv Q|∼X}$, if *P* believes that s/he shared the key *K* with *Q*, and *P* receives the message ${\left[X\right]}_{k}$; then, *P* believes that *Q* sent *X*.

**R2 (Belief rule).**$\frac{P|\equiv Q|∼(X,Y)}{P|\equiv Q|∼X}$, if *P* believes *Q* sends the message set (X,Y); then, *P* believes *Q* sends the message *X*.

Our formal security analysis is split into the following steps:

**Step 1. Protocol messages.**

**PM1:** $MI_{i},N_{i},P_{i},Q_{i},L_{i},T_{1},$

**PM2:** $N_{j},SS_{j},V_{j},T_{2},$

**PM3:** $W_{j},K_{i}j,T_{3},$

**PM4:** $M_{1},K_{i}j,T_{4},$

**Step 2. Idealizing the protocol messages.** At this point, the protocol messages are converted into the idealized format based on the BAN-logic notations. The results are denoted by IM1, ..., IM9 as below:

**IM1 ($U_{i}\to \mathrm{GWN}$):** $GWN◃{\left\{K_{i}\right\}}_{h(MI_{i}∥X_{GWN})},$

**IM2 ($U_{i}\to \mathrm{GWN}$):** $GWN◃{\left\{SID_{j}\right\}}_{h(MI_{i}∥X_{GWN})},$

**IM3 ($U_{i}\to \mathrm{GWN}$):** $GWN◃{\left(MI_{i},K_{i},T_{1},SID_{j},Q_{i}\right)}_{h(MI_{i}∥X_{GWN})},$

**IM4 ($U_{i}\to \mathrm{GWN}$):** $GWN◃{\left\{h\left(ID_{i}\right)\right\}}_{K_{i}},$

**IM5 ($\mathrm{GWN}\to S_{j}$):** $S_{j}◃{\left(h\left(ID_{i}\right),T_{2},K_{i}\right)}_{h(SID_{j}∥X_{GWN})},$

**IM6 ($S_{j}\to \mathrm{GWN}$):** $GWN◃{\left\{K_{j}\right\}}_{K_{i}},$

**IM7 ($S_{j}\to \mathrm{GWN}$):** $GWN◃{\left(SK_{j}\right)}_{T_{3}},$

**IM8 ($\mathrm{GWN}\to U_{i}$):** $U_{i}◃{\left\{K_{j}\right\}}_{K_{i}},$

**IM9 ($\mathrm{GWN}\to U_{i}$):** $U_{i}◃{\left(SK_{G}\right)}_{K_{j}}.$

**Step 3. Explicit assumptions.** The seven assumptions on the proposed scheme are described by A1, ..., A7 as below:

**A1:** $U_{i}|\equiv ♯\left(K_{i},T_{1},T_{4}\right),$

**A2:** $GWN|\equiv ♯(T_{1},T_{2},T_{3},T_{4}),$

**A3:** $S_{j}|\equiv ♯\left(K_{j},T_{2},T_{3}\right),$

**A4:** $U_{i}|\equiv U_{i}\overset{h(MI_{i}∥X_{GWN})}{⟷}GWN,$

**A5:** $GWN|\equiv GWN\overset{h(MI_{i}∥X_{GWN})}{⟷}U_{i},$

**A6:** $GWN|\equiv GWN\overset{h(SID_{j}∥X_{GWN})}{⟷}S_{j},$

**A7:** $S_{j}|\equiv S_{j}\overset{h(SID_{j}∥X_{GWN})}{⟷}GWN.$

**Step 4. Security goals.** The nine security goals which are expected to be verified after analyzing the protocol by BAN-logic are listed by G1, ..., G9 as below. For instance, the goal G1 states that the gateway node must believe that the user $U_{i}$ has sent the key $K_{i}$:

**G1:** $GWN|\equiv U_{i}|∼K_{i},$

**G2:** $GWN|\equiv U_{i}|∼SID_{j},$

**G3:** $GWN|\equiv U_{i}|∼h\left(ID_{i}\right),$

**G4:** $S_{j}|\equiv GWN|∼K_{i},$

**G5:** $S_{j}|\equiv GWN|∼h\left(ID_{i}\right),$

**G6:** $GWN|\equiv S_{j}|∼K_{j},$

**G7:** $GWN|\equiv S_{j}|∼SK_{j},$

**G8:** $U_{i}|\equiv GWN|∼K_{j},$

**G9:** $U_{i}|\equiv GWN|∼SK_{G}.$

**Step 5. Deriving the security goals.** Finally, to show the achievement of the above-mentioned goals, we apply logical rules of the BAN-logic to the idealized messages and initial premises as described below.

In accordance with IM1, A5 and R1:

**Result1:**$GWN|\equiv U_{i}|∼K_{i}$ (satisfy **G**1);

Given the IM2, A5 and R1:

**Result2:**$GWN|\equiv U_{i}|∼SID_{j}$ (satisfy **G**2);

In accordance with IM4, Result1 and R1:

**Result3:**$GWN|\equiv U_{i}|∼h{\left(ID\right)}_{i}$ (satisfy **G**3);

Given the IM5, A7 and R1:

**Result4:** $S_{j}|\equiv GWN|∼\left(h\left(ID_{i}\right),T_{2},K_{i}\right);$

Taking into account Result4 and R2:

**Result5:**$S_{j}|\equiv GWN|∼K_{i}$ (satisfy **G**4);

**Result6:**$S_{j}|\equiv GWN|∼h\left(ID_{i}\right)$ (satisfy **G**5);

In accordance with IM6, A6 and R1:

**Result7:**$GWN|\equiv S_{j}|∼K_{j}$ (satisfy **G**6);

In accordance with IM7, A2 and R1:

**Result8:**$GWN|\equiv S_{j}|∼SK_{j}$ (satisfy **G**7);

In accordance with IM8, A1 and R1:

**Result9:**$U_{i}|\equiv GWN|∼K_{j}$ (satisfy **G**8);

In accordance with IM9, Result9 and R1:

**Result10:**$U_{i}|\equiv GWN|∼SK_{G}$ (satisfy **G**9).

Given the above steps, it can easily be concluded that the protocol can meet all preset goals. Therefore, we can state that our proposed scheme is secure.

## 8. Performance Comparison

In this work, we propose a new 3FA protocol to overcome the security weaknesses of the Amin et al. [15] scheme. We show how our enhanced protocol is not only secure but also efficient enough to be used in HWSNs. The discussion about the security features, computational overhead and computational cost offered by our proposed scheme and other related schemes, such as Amin et al. [15], Yeh et al. [32], Xue et al. [7], Das et al. [44], Jiang et al. [33], Das et al. [45] and Gope et al. [24] is presented in this section.

### 8.1. Security Features’ Comparison

In Table 3, we sum up the security features offered by our proposed protocol and other similar ones. The symbol “Yes” indicates that the scheme is secure against the related attack and the symbol “No” indicates the contrary. From this, we can conclude that our proposal satisfies all the security features required and offers a higher security level than its predecessors. In addition, protocols [7,24,32,33] do not provide three-factor authentication while our scheme does.

### 8.2. Overall Computational Overhead Comparison

In HWSNs, sensors have limited energy so any authentication protocol designed for these networks should be lightweight and energy efficient. Moreover, we use the model represented in Figure 1a to design our scheme. In our scheme, we use the hash, and the fuzzy extractor functions, which are both efficient. In fact, using the low-power cryptographic functions, rather than a very demanding one, can reduce energy consumption [46]. According to the results of the experiments presented in [24], each modular exponential operation in ECC-160 algorithm consumes 1.2 Ws energy and takes $t_{Exp}$ = 11.69 ms execution time. Moreover, for symmetric key encryption/decryption (128-bit AES-CBC), the running time and energy consumption are approximately $t_{sym}$ = 4.62 ms and 0.72 Ws and for hash function (SHA-256) these two values are approximately $t_{Hash}$ = 1.06 ms and 0.27 Ws, respectively. These results were obtained using the MSB-430 sensor boards with the TI MSP430 micro controller [24]. Moreover, the time that the fuzzy extractor takes $t_{f}$ is about 17.1 ms [47]. In Table 4, previous works [7,15,24,32,33,44,45] and our proposed scheme are compared in terms of computational cost. As shown in this table, in our proposal, the total computational cost is only $25\times t_{Hash}+t_{f}$. Although our proposed scheme consumes slightly more time than some proposals [7,24,33], these extra time is because of the additional operations needed for securing the scheme (improving security pitfalls of its predecessors) and the three-factor capability, which is critical for secure HWSN networks. Finally, it is worth noticing that our results are similar to [15,45], but we offer a higher security level.

### 8.3. Computational Cost and Execution Time

To achieve better efficiency and taking into account the energy restrictions of sensor nodes, the computation costs of sensors should be kept as low as possible. In Table 5, we summarize both the computational cost and execution time of our proposal and its predecessors [7,15,32,33,44,45]. From this, it is clear that our proposal is one of the most efficient in terms of energy and execution time. That is, our proposal can be fitted in resource-limited sensor nodes.

## 9. Conclusions

In heterogeneous wireless sensor networks (HWSNs), we find sensors with different capabilities and functionalities and dispersed within a defined area. Generally, their capabilities, such as computation and energy, are very limited. The security of these devices is pivotal and challenging due to its constrained resources. In this vein, we propose a secure and efficient three-factor authentication (3FA) scheme that is suitable for HWSNs and enhances the security of a recent proposed protocol [15]. Meanwhile, we showed how [15] is not resistant to user impersonation and de-synchronization attacks and also the attacker can track the user by eavesdropping only one session. In addition, an adversary can disclose the session key under the common assumption that the hardware of sensors is not tamper-resistant. To scrutinize the security of our proposal, we informally and formally analyze its security and show how our protocol guarantees all the security features and provides the highest security level in comparison with their predecessors. Moreover, in relation to performance, our scheme consumes only few milliseconds and is very efficient in terms of energy consumption. All of this renders our scheme adequate for HWSNs in which sensors generally have very limited resources. Therefore, as a future work, we aim to propose a new scheme to support user access control that guarantees authorized users to access the information allowed in HWSNs. 

## Figures and Tables

**Table 1:** Notations.

Notation	Description
$U_{i}$	The *i*-th user
GWN	The gateway node
$SC_{i}$	The smartcard of $U_{i}$
$S_{j}$	The *j*-th sensor node
$Z_{q}^{*}$	Multiplicative group, where q is a large prime,
	$Z_{q}^{*}=\left\{x:0<x<q,gcd\left(x,q\right)=1\right\}$
$ID_{i}$	Identity of $U_{i}$
$SID_{j}$	Identity of $S_{j}$
$X_{GWN}$	Secret key of GWN
$f_{i}$	Secret key linked to $U_{i}$
$f_{j}$	Secret key linked to $S_{j}$
$PW_{i}$	Password linked to $U_{i}$
$B_{i}$	Biometric trait linked to $U_{i}$
$K_{i}$	Nonce generated by $U_{i}$
$K_{j}$	Nonce generated by $S_{j}$
$SK_{i},SK_{j},SK_{G}$	Session key
REP(·),GEN(·)	Fuzzy extractor operations
$\psi_{i},\theta_{i}$	Outputs of GEN(·) algorithm
$T_{i}$	Timestamp
ΔT	Allowable transmission delay
h(·)	One-way hash function
⊕	Bitwise XOR operation
||	Concatenation operation

**Table 2:** BAN-logic notations.

Notation	Description
P∣≡X	*P* believes a proposition *X*
P◃X	*P* receives a message *X*
P∣∼X	*P* sent a message *X*
$P\overset{k}{⇌}X$	*P* and *X* share the secret key *k* and only these two entities can use *k* to prove its identity to each other.
♯(X)	It means that *X* is fresh
${\left\{X\right\}}_{k}$	Encryption of *X* using the secret *k*
${\left(X\right)}_{k}$	Hash computation of *X* using the secret *k*
$P\overset{k}{↔}Q$	*P* and *Q* share a secret *k*
$\frac{P}{Q}$	If *P* then *Q*

**Table 3:** Security features’ comparison.

Security Features	Amin et al. [15]	Yeh et al. [32]	Xue et al. [7]	Das [44]	Jiang et al. [33]	Das [45]	Gope et al. [24]	Ours
Protection of user untraceability	No	No	No	Yes	Yes	Yes	No	**Yes**
Resistance against replay attack	Yes	No	Yes	Yes	Yes	Yes	Yes	**Yes**
Resistance against user impersonation attack	No	No	No	Yes	No	Yes	Yes	**Yes**
Resistance against gateway node impersonation attack	Yes	No	No	No	No	No	Yes	**Yes**
Resistance against sensor node impersonation attack	Yes	Yes	Yes	Yes	Yes	Yes	Yes	**Yes**
Resistance to de-synchronization attack	No	No	No	No	No	No	Yes	**Yes**
Support of dynamic node addition	Yes	No	No	Yes	No	Yes	Yes	**Yes**
Robustness against insider attack	Yes	Yes	No	Yes	No	Yes	Yes	**Yes**
Robustness against stolen smartcard attack	Yes	No	No	Yes	No	Yes	Yes	**Yes**
User anonymity	Yes	No	No	No	Yes	Yes	Yes	**Yes**
Resistance against identity guessing attack	Yes	No	No	Yes	Yes	Yes	Yes	**Yes**
Support of three-factor security	Yes	No	No	Yes	No	Yes	No	**Yes**
Supports correct password update	Yes	No	No	Yes	No	Yes	No	**Yes**
Resistance against session key disclosure attack	No	Yes	Yes	Yes	Yes	Yes	No	**Yes**

**Table 4:** Overall computational overhead of the authentication phase.

Scheme	User	GW	Sensor Node	Total Cost	Rough Estimation
Amin et al. [15]	$10t_{Hash}+t_{f}$	$11t_{Hash}$	$4t_{Hash}$	$25t_{Hash}+t_{f}$	43 ms
Yeh et al. [32]	$2t_{Exp}+t_{Hash}$	$4t_{Exp}+4t_{Hash}$	$2t_{Exp}+3t_{Hash}$	$8t_{Hash}+8t_{Exp}$	100 ms
Xue et al. [7]	$7t_{Hash}$	$10t_{Hash}$	$5t_{Hash}$	$22t_{Hash}$	23 ms
Das [44]	$7t_{Hash}+t_{f}$	$t_{Sym}+2t_{Hash}$	$t_{Sym}+2t_{Hash}$	$11t_{Hash}+2t_{Sym}+t_{f}$	38 ms
Jiang et al. [33]	$7t_{Hash}$	$10t_{Hash}$	$5t_{Hash}$	$22t_{Hash}$	23 ms
Das [45]	$9t_{Hash}+t_{f}$	$11t_{Hash}$	$5t_{Hash}$	$25t_{Hash}+t_{f}$	43 ms
Gope et al. [24]	$7t_{Hash}$	$9t_{Hash}$	$3t_{Hash}$	$19t_{Hash}$	20 ms
Ours	$10t_{Hash}+t_{f}$	$11t_{Hash}$	$4t_{Hash}$	$25t_{Hash}+t_{f}$	43ms

**Table 5:** Computational cost and execution time comparison.

Scheme	Computational Cost	Execution Time
Amin et al. [15]	1.08 Ws	4.24 ms
Yeh et al. [32]	3.21 Ws	26.56 ms
Xue et al. [7]	1.35 Ws	5.3 ms
Das [44]	1.53 Ws	7.8 ms
Jiang et al. [33]	1.35 Ws	5.3 ms
Das [45]	1.35 Ws	5.3 ms
Gope et al. [24]	0.81 Ws	3.18 ms
Ours	1.08 Ws	4.24 ms
